# Advances and challenges in neoantigen prediction for cancer immunotherapy

**DOI:** 10.3389/fimmu.2025.1617654

**Published:** 2025-06-12

**Authors:** Yi Zhang, Ting-Ting Chen, Xiong Li, Ai-Lin Lan, Peng-Fei Ji, Ya-Juan Zhu, Xue-Yao Ma

**Affiliations:** ^1^ The First Clinical Medical College of Lanzhou University, Lanzhou, China; ^2^ Department of Gastroenterology, The Second Hospital & Clinical Medical School, Lanzhou University, Lanzhou, China; ^3^ Department of Gastroenterology, The Second Affiliated Hospital of Xi’an Jiaotong University, Xi’an, China; ^4^ Department of Thoracic Surgery, West China Hospital, Sichuan University, Chengdu, China; ^5^ Department of Biotherapy and Cancer Center, State Key Laboratory of Biotherapy, West China Hospital, Sichuan University, Chengdu, China; ^6^ Department of Gynecology, The Second Hospital & Clinical Medical School, Lanzhou University, Lanzhou, China

**Keywords:** neoantigen prediction, cancer immunotherapy, next-generation sequencing, immunopeptidomics, bioinformatics, immune evasion

## Abstract

Neoantigens, derived from tumor-specific mutations, are promising targets of cancer immunotherapy by eliciting tumor-specific T-cell responses while sparing normal cells. Accurate neoantigen prediction relies on immunogenomics and immunopeptidomics. Immunogenomics identifies tumor-specific mutations via next-generation sequencing. Immunopeptidomics detects MHC-presented peptides using mass spectrometry. Integrating these two methods enhances prediction accuracy but faces challenges due to tumor heterogeneity, HLA diversity, and immune evasion. Future advancements will focus on dynamic tumor microenvironment monitoring, multi-omics integration, improved computational models and algorithms to refine neoantigen prediction, and developing optimized personalized vaccines.

## 1 Introduction

The landscape of cancer therapy is undergoing a transformative shift from traditional chemotherapy and radiotherapy to immunotherapy, which focuses on modulating the tumor microenvironment to activate the patient’s immune system (1). The advent of immune checkpoint inhibitors (ICIs), such as PD-1 and CTLA-4, heralds a new era in cancer immunotherapy (2, 3). Despite their ability to elicit durable clinical responses against various malignancies, the efficacy of these inhibitors is not universally observed across patient populations (4–7).

Neoantigens, arising from unique mutations in tumor cells, are expressed solely within the tumor (8), capable of eliciting T-cell responses against the tumor with minimal immune tolerance (9, 10). These neoantigens typically originate from genetic mutations in tumor cells, such as single nucleotide variations, gene rearrangements, and splicing alterations, making them ideal targets for immunotherapy (11). The concomitant administration of ICIs alongside neoantigen-based immunotherapies represents a synergistic strategy to enhance the efficacy of tumor-directed T cell responses (12). The ongoing investigation of neoantigens as biomarkers for immunotherapy response is expected to refine patient stratification protocols (6, 13), thereby enhancing the precision of therapeutic interventions and enabling personalized treatment regimens to maximize clinical benefits.

With the rapid advancement of next-generation sequencing technologies like Whole Genome Sequencing (WGS) and Whole Exome Sequencing (WES), the identification of genomic variations in tumors has become significantly more efficient (14, 15). However, predicting the immunogenicity of these variations remains a major challenge (10). Simultaneously, mass spectrometry (MS) is used to directly identify peptides that actually exist in tumor cells, aiding in the detection of neoantigens that might be lost due to immune escape or overlooked due to their rarity or low abundance in traditional bioinformatics predictions (16, 17).

Despite these advancements, neoantigen prediction and validation continue to face substantial challenges. Immune evasion further complicates accurate prediction, underscoring the need for precise strategies and combination therapies to enhance immunotherapy efficacy. Key areas for improvement include refining MHC-peptide binding predictions, expanding datasets, and developing more advanced algorithms—crucial steps toward achieving more precise and effective cancer immunotherapy.

## 2 The biological mechanism of neoantigens

**Figure 1:**
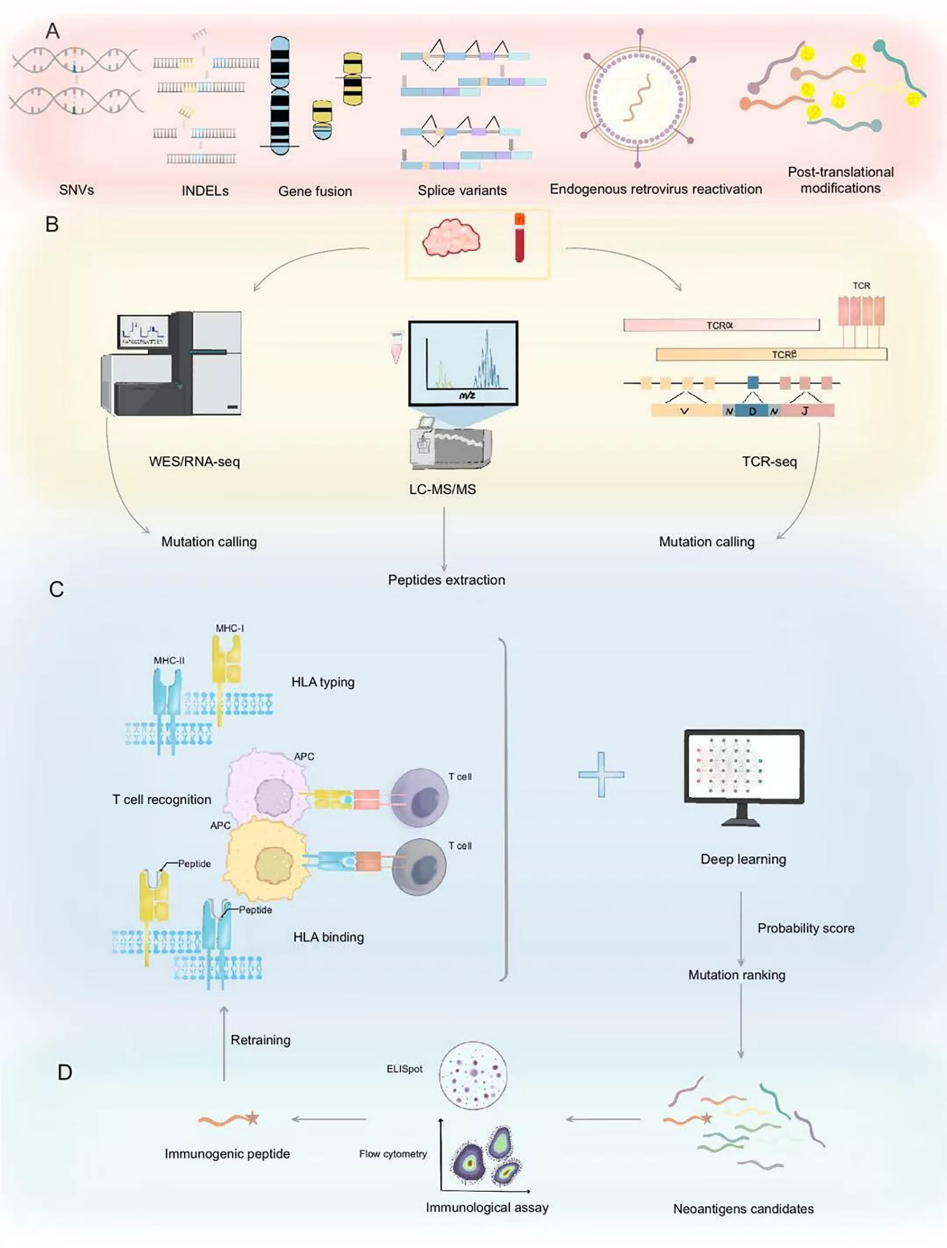
Neoantigen prediction and validation workflow. **(A)** Neoantigens originate from diverse genetic alterations including SNVs, INDELs, gene fusions, splice variants, endogenous retroviruses, and post-translational modifications. **(B)** Neoantigen prediction initiates with mutation identification through WES/RNA-seq and peptide extraction via LC-MS/MS, complemented by TCR-seq for immune receptor profiling. **(C)** The process begins with HLA typing of the peptides to assess their binding affinity to HLA molecules. This step is crucial for identifying which peptides can be recognized by the immune system. Following this, the peptides undergo T cell recognition assays to evaluate their ability to activate T cells. Deep learning models are then employed to predict the presentation probability of neoantigens and to rank mutations, thereby prioritizing the most promising candidate peptides. These models analyze extensive datasets, including peptide sequences, HLA types, and expression levels, to forecast the immunogenicity of the peptides. **(D)** Validated immunogenic peptides are confirmed through immunological assays, identifying potential neoantigen candidates for cancer immunotherapy. Validated neoantigen data can serve as input for retraining, which is used to refine and enhance the predictive accuracy of deep learning models. SNVs, Single Nucleotide Variants; INDELs, Insertions and Deletions; WES/RNA-seq, Whole Exome/RNA sequencing; LC-MS/MS, Liquid Chromatography-Mass Spectrometry/Mass Spectrometry; TCR, T-cell receptor; MHC, Major Histocompatibility Complex; APC, Antigen-Presenting Cell; HLA, Human Leukocyte Antigen.

Neoantigens are tumor-specific protein fragments generated by cancer cell mutations, exhibiting minimal structural similarity to normal proteins, and rarely shared among patients. Unlike tumor-associated antigens, neoantigens offer high tumor specificity, reducing off-target toxicity and addressing antigen scarcity in targeted cancer immunotherapy (10, 11, 18). Neoantigens arise from various mechanisms, including non-synonymous single-nucleotide variants (SNVs), insertions/deletions (INDELs), gene fusions, splice variants, endogenous retrovirus reactivation, and protein fragments generated by HLA somatic mutations or non-coding region translation (19, 20) (**Figure 1**). The high specificity of neoantigens makes them attractive biomarkers for immunotherapy response. They can serve as targets for personalized cancer vaccines and adoptive T-cell therapies (21). Furthermore, the load and quality of neoantigens have been linked to patient outcomes in several malignancies, including metastatic melanoma and non-small cell lung cancer (22, 23). However, only a small fraction of SNVs is immunogenic (24), limiting their vaccine potential to cancers with a high neoantigen load, such as metastatic melanoma with high SNV burden (20, 25). Their low prevalence across patients poses challenges for the development of universal therapeutic approaches. Leukemias and sarcomas, which have lower SNV burden, often express shared gene fusions and splice variant transcripts, indicating the potential for universal therapy development (14). The new tool SNAF systematically identifies splicing neoantigens, revealing potential common targets in the treatment of heterogeneous cancers (25). Thus, accurately identifying and validating neoantigens is a crucial strategy for optimizing immunotherapy applications. Based on the different sources of neoantigens, various identification methods are employed. **Table 1** summarized three strategies for the identification of neoantigens.

**Table 1:** Overview of neoantigen epitope identification strategies.

Identification strategy	Description	Technological methods	Advantages	Limitations
Immunogenomic Strategy	Predicts neoantigens based on NGS data using computational methods	·Whole Exome Sequencing (WES) ·RNA Sequencing (RNA-seq) ·Computational prediction tools (e.g., NetMHC, MHCflurry)	·Rapid screening of a large number of potential neoantigens ·Suitable for large-scale analysis	·Requires experimental validation ·Possibility of false positives
Immunopeptidomic Strategy	Analyzes MHC-loaded peptides using mass spectrometry	·Mass Spectrometry (MS) ·Immunoprecipitation (IP)	·Provides direct experimental evidence ·Identifies peptides actually presented on tumor cells	·Limited by the coverage and sensitivity of mass spectrometry technology ·High cost and complexity of operation
TCR-Guided Strategies	Utilizes TCR specificity to identify neoantigens	·TCR transgenic mouse models ·TCR-cloned T cells ·TCR-mimic antibodies	·Directly assesses the immunogenicity of neoantigens ·Suitable for functional validation	·Requires specific TCR tools ·May be limited by the availability of TCRs

## 3 Technological platforms for neoantigen prediction

### 3.1 Genomic and transcriptomic approaches

Neoantigens arise from five primary sources: genomic variations, transcriptomic variations, proteomic variations, virus-derived tumor antigens, and other sources (11). Genomic variations include SNVs, INDELs, and gene fusions. (Next-Generation Sequencing) NGS technologies, such as Whole Genome Sequencing (WGS) and Whole Exome Sequencing (WES), have enabled the rapid identification of genomic alterations in tumors. By comparing tumor and normal tissue sequencing data, researchers can pinpoint somatic mutations that may lead to neoantigen formation. Complementary RNA sequencing (RNA-Seq) further helps in assessing the expression levels of these mutant genes and detecting alternative splicing events (26). By analyzing sequencing data from individual tumors, neoantigens derived from SNVs and INDELs can be identified as potential target epitopes. Conceptually, predicting epitopes based on NGS data involves three steps: (a) converting genomic mutations into mutated protein sequences, (b) predicting MHC binding, and (c) evaluating immunogenicity based on predicted binding, expression levels, and sequence similarity to non-mutated self-proteins (27) (**Figure 1**). These steps reflect the core principle that an immune response requires the transcription, translation, processing, and MHC presentation of abnormal gene products. The integration of genomic and transcriptomic data is critical for filtering potential neoantigen candidates. Consequently, verifying expression and assessing MHC-binding affinity are pivotal in current computational pipelines (9). For example, the automated workflow pVAC-Seq (28), developed by J. Hundal et al., evaluates the binding capacity of candidate neoantigens to a patient’s HLA molecules, integrating tumor gene expression and mutation coverage data. Tools such as BWA (Burrows-Wheeler Aligner) and GMS (Genome Modeling System) align WES/WGS and RNA-seq data, while HLAminer and Athlate perform HLA typing. Mutations are annotated using the Ensembl database and Variant Effect Predictor (VEP), and NetMHC (29) predicts binding affinities, followed by filters to select optimal mutated peptide candidates. Similar pipelines include TSNAD (30), CloudNeo (31), and TIminer (32). Although these methods consider MHC-peptide affinity, they often omit other crucial factors like TCR recognition and sequence similarity to highly immunogenic epitopes (33, 34). In contrast, pipelines such as MixMHCpred2.2 and PRIME2.0 (35), address these limitations, improving predictions of antigen presentation and TCR recognition.

### 3.2 Immunopeptidomics

Immunopeptidomics uses mass spectrometry (MS) to directly identify peptides presented by MHC molecules in tumor cells. This approach involves two steps: (1) isolating MHC-bound peptides from tumor cells and (2) analyzing these peptides via MS to pinpoint tumor-specific neoantigens (36) (**Figure 1**). MS-based immunopeptidomics has emerged as a powerful tool to directly profile the peptides presented on MHC molecules. This method complements NGS by verifying the actual presence of neoantigens on tumor cells (37). The direct detection of MHC-bound peptides addresses some limitations of bioinformatic predictions, particularly for neoantigens that are rare or prone to immune escape (38). To improve the positive predictive value of neoantigen prediction, advanced models integrate deep learning with MS data. The EDGE model (39) is trained directly using MS data rather than HLA-peptide binding affinity measurements *in vitro*. SHERPA (40) systematically combines 128 monoallelic and 384 multiallelic samples, employing an HLA-null K562 parental cell line with stably transfected HLA alleles to better emulate native antigen presentation. However, proteomics-based approaches have their own drawbacks, including limited sensitivity in detecting low-abundance peptides, technical variability in MS data, and the inability to capture all potential neoantigens due to sample processing constraints (**Table 1**).

### 3.3 Bioinformatics and Computational Models

Bioinformatics tools play a pivotal role in predicting neoantigen immunogenicity. Several algorithms, including RPEMHC (41), UniPMT (42) and TEIM-Res (43) have been developed to assess MHC-peptide binding affinity, stability, and the likelihood of TCR recognition, respectively. Machine learning and deep learning further improved prediction accuracy by integrating large-scale multi-omics datasets, such as proteomic and immunopeptidomic profiles (44). However, the variability in HLA alleles and the complexity of antigen processing pathway continue to pose challenges for these computational models (45).

## 4 Challenges in neoantigen prediction

### 4.1 Tumor heterogeneity

The inherent heterogeneity of tumors, both spatially and temporally, complicates the accurate prediction of neoantigens. Variability in mutation profiles within a single tumor and across different tumor sites may lead to inconsistent neoantigen expression and immune recognition. The analysis of melanoma samples from two distinct metastatic sites—the gastrointestinal tract and the pelvic cavity—in the same patient revealed significant heterogeneity in the functionality of the antigen presentation machinery (APPM) (44). Some tumor cells lose their ability of antigen presentation due to the deletion or mutation of β2-microglobulin (β2M) gene (46). This highlights the importance of considering tumor spatial heterogeneity when predicting neoantigens. By integrating multi-omics data, NeoDisc (44) can identify the defects in the mechanism of antigen presentation, shaping the heterogeneous landscape of tumor antigens. Over time, certain neoantigens may no longer be effectively presented due to the tumor evolution (47), or their immunogenicity may diminish as a results of alterations in the tumor microenvironment (48). To better understand the temporal heterogeneity of tumors, analyzing tumor samples at different time points provides valuable insights into their evolution.

### 4.2 Tumor microenvironment complexity

The immunogenicity of neoantigens is shaped by both their intrinsic physicochemical properties, such as MHC-binding affinity and T cell receptor recognition and the dynamic interplay within the tumor microenvironment (TME). Neoantigens from mutant proteins at the cell membrane are more accessible to immune cells, enhancing immunogenicity, whereas those confined to the nucleus or other organelles are less likely to be processed and presented effectively (49).

Within the TME, immunosuppressive cells play a critical role. Regulatory T cells (Tregs) express high levels of CTLA-4 and PD-1, suppressing effector T cell activity (50). Myeloid-derived suppressor cells (MDSCs) secrete inhibitory factors such as IL-10, TGF-β, and arginase, further attenuating T cell responses (51). Tumor-associated macrophages (TAMs) also contribute by releasing cytokines like IL-10, which impair dendritic cell maturation and antigen presentation (52). TME exhibits considerable heterogeneity across different tumor types and individuals, which significantly influences the immunogenicity of neoantigens (53). Moreover, the composition of the TME varies significantly among tumors, with some dominated by Tregs and others by MDSCs, underscoring the need for personalized neoantigen prediction models that account for these differences. Combining TME remodeling strategies with immunotherapies—such as pairing ICIs with chemotherapy or targeted therapies—has improved clinical outcomes (52, 54). To achieve this, it is critical to incorporate TME remodeling strategies into neoantigen prediction frameworks, enabling more precise and tailored immunotherapies. This necessitates a holistic understanding of TME complexity, leveraging multi-omics data from genomics, transcriptomics, proteomics, and metabolomics. Furthermore, advanced simulations of the TME during neoantigen screening are vital to assess the immunogenicity of neoantigens within their specific heterogeneous contexts. For example, integrating the functional profiles of immunosuppressive cell populations, such as Tregs and MDSCs, into predictive models can refine neoantigen selection. These evolving requirements underscore the growing challenges and opportunities in developing robust neoantigen prediction pipelines.

### 4.3 HLA polymorphism

The high degree of HLA polymorphism among individuals adds another layer of complexity. The HLA gene is situated on the short arm of human chromosome 6 and represents one of the most complex gene systems in the human genome (55). Due to the polymorphism of HLA gene, there are significant differences in genetic background among different races and regions (56). This polymorphism is primarily manifested in the amino acid positions of the antigen-binding groove, where variations at these sites determine the specific antigenic peptides that HLA molecules can bind, as well as the peptide binding affinity, stability and TCR recognition ability (57). Differences in HLA binding preferences and antigen presentation can significantly affect the immunogenicity of predicted neoantigens, necessitating personalized approaches in prediction and validation.

### 4.4 Limitations of current algorithms and data

The performance of current bioinformatics models is inherently constrained by the quality and size of available datasets, which directly influence their predictive accuracy and generalizability. Incomplete datasets with lacking essential genomic, transcriptomic, or proteomic information, pose significant challenges to comprehensive characterization of neoantigens (58). Furthermore, many models are trained on datasets that lack sufficient representation across diverse cancer types, potentially introducing biases and reducing their applicability to underrepresented malignancies (33). These limitations often result in overfitting, where models exhibit high performance on training data but fail to generalize to independent datasets, or result in misclassification of neoantigen immunogenicity, producing false-positive or false-negative predictions that may impact therapeutic decision-making (59). To overcome these challenges, efforts are required to enhance dataset curation, including the integration of multi-omics data, the establishment of standardized data collection protocols, and the inclusion of diverse patient cohorts (60). Additionally, the complexity of algorithmic models presents difficulties in interpretability, which may compromise their reliability in both research and clinical settings. Improving model transparency and developing explainable AI frameworks could mitigate these issues, ultimately fostering greater confidence in computational predictions (61).

## 5 Future perspectives

Accurate neoantigen prediction is essential for the development of personalized cancer vaccines and adoptive T-cell therapies. Tailoring immunotherapy to a patient’s unique mutational landscape holds great potential for enhancing treatment efficacy while minimizing adverse effects. However, several challenges remain in improving the precision and applicability of neoantigen-based therapeutic strategies.

A comprehensive approach to neoantigen prediction involves multiple advancements. First, employing longitudinal sampling and single-cell sequencing enables the capture of tumor evolution and neoantigen dynamics over time. Second, integrating multi-omics data with advanced computational models can significantly enhance prediction accuracy. Third, developing more sophisticated algorithms that account for HLA polymorphism and tumor immune evasion mechanisms is crucial for improving the reliability of neoantigen identification. Additionally, establishing standardized protocols for validating predicted neoantigens in clinical trials is imperative to ensure their translational success.

The incorporation of artificial intelligence and machine learning into neoantigen prediction pipelines presents a promising avenue for future research. By enhancing predictive models and enabling real-time monitoring of tumor immunogenicity, these technologies may contribute to the development of more effective and adaptive cancer immunotherapy regimens. Furthermore, combining neoantigen-based strategies with other immunotherapies, such as immune checkpoint inhibitors, has the potential to synergistically enhance anti-tumor responses. Such combination approaches could help counteract immune evasion mechanisms and ultimately improve clinical outcomes for cancer patients.

## 6 Conclusion

Neoantigen prediction is at the forefront of personalized cancer immunotherapy. Despite significant technological advances, challenges such as tumor heterogeneity, HLA variability, and immune evasion remain. Continued innovation in sequencing technologies, immunopeptidomics, and computational models is essential for overcoming these obstacles. Future research that bridges the gap between prediction and clinical validation will be critical for translating neoantigen-based strategies into routine clinical practice.
