# Supplementary material for: Advances and challenges in neoantigen prediction for cancer immunotherapy
Source: Front Immunol. 2025 Jun 12;16:1617654. doi: 10.3389/fimmu.2025.1617654 (PMC12198247; doi:10.3389/fimmu.2025.1617654)
Supplement: Supplementary file 1 [file Table1.docx]

**Supplementary Tabel.** **Comparison of Neoantigen Prediction Pipelines and Tools**

| **Name** | **Neoantigen types** | **Input data** | **Neoantigen class** | **Comment** |
| --- | --- | --- | --- | --- |
|  |  |  |  |  |
| [NUCC](https://doi.org/10.1080/21645515.2023.2300881)(1)^*^ | SNVs, INDELs | RNA-seq, WES, HLA types | Class I | Based on deep learning (CNN and FCNN); integrating peptide sequences, HLA types, binding affinity, and stability; outperforming NetMHCpan in identifying neoantigens; validated in gastric cancer patients. |
| [LRMAHpan](https://www.frontiersin.org/journals/immunology/articles/10.3389/fimmu.2024.1478201/full)(2)^*^ | SNVs,  INDELs, gene fusions | MS data, HLA types | Class I and II | Based on ResNet and LSTM networks; integrating multi-channel matrices and peptide sequences; better than NetMHCpan, MHCflurry; high-quality multi-allele MS data is required |
| [TCRβ-Neoantigen Prediction](https://elifesciences.org/articles/94658)(3) | SNVs,  INDELs, gene fusions | TCRβ sequencing data, HLA types, RNA-seq | Class I and II | Combining TCRβ sequencing data and pHLA binding prediction; integrating pHLA and pHLA-TCR binding information through ML; excellent in colorectal cancer; limited to specific cancer species |
| [SHERPA](https://www.sciencedirect.com/science/article/pii/S1535947623000154?via%3Dihub)(4) | SNVs, INDELs, gene fusions | MS data, HLA types | Class I | Integrating large-scale immunopeptidomics data and composite modeling; outperforming existing tools in predicting MHC binding and presentation; validated in monoallelic and multiallelic datasets. |
| [PGNneo](https://www.mdpi.com/2073-4409/12/5/782)(5) | SNVs, INDELs in noncoding regions | RNA-seq, MS data, HLA types | Class I | Focusing on noncoding regions; combining proteogenomics data to reduce false positives; validated in hepatocellular carcinoma and colorectal cancer cohorts. |
| [Neodb](https://academic.oup.com/database/article/doi/10.1093/database/baad041/7197208)(6) | SNVs, INDELs | HLA types, mutation data | Class I and II | Comprehensive database and discovery platform; including experimentally validated neoantigens and a novel GNN-based immunogenicity prediction tool (Immuno-GNN); supporting multiple cancer types. |

Note: * are tools to improve the accuracy of neoantigen prediction, and the remaining are comprehensive neoantigen pipelines for prediction. Among them, PGNneo uses immunogenomic strategy, SHERPA uses immunopeptidomic strategy, TCRβ-Neoantigen Prediction uses TCR-Guided strategies.

1. Xin K, Wei X, Shao J, Chen F, Liu Q, Liu B. Establishment of a novel tumor neoantigen prediction tool for personalized vaccine design. Hum Vaccin Immunother. 2024;20(1):2300881.

2. Mi X, Li S, Ye Z, Dai Z, Ding B, Sun B, et al. LRMAHpan: a novel tool for multi-allelic HLA presentation prediction using Resnet-based and LSTM-based neural networks. Front Immunol. 2024;15:1478201.

3. Pham TMQ, Nguyen TN, Tran Nguyen BQ, Diem Tran TP, Diem Pham NM, Phuc Nguyen HT, et al. The T cell receptor β chain repertoire of tumor infiltrating lymphocytes improves neoantigen prediction and prioritization. Elife. 2024;13:RP94658.

4. Pyke RM, Mellacheruvu D, Dea S, Abbott C, Zhang SV, Phillips NA, et al. Precision Neoantigen Discovery Using Large-Scale Immunopeptidomes and Composite Modeling of MHC Peptide Presentation. Mol Cell Proteomics. 2023;22(4):100506.

5. Tan X, Xu L, Jian X, Ouyang J, Hu B, Yang X, et al. PGNneo: A Proteogenomics-Based Neoantigen Prediction Pipeline in Noncoding Regions. Cells. 2023;12(5):782.

6. Wu T, Chen J, Diao K, Wang G, Wang J, Yao H, et al. Neodb: a comprehensive neoantigen database and discovery platform for cancer immunotherapy. Database (Oxford). 2023;2023:baad041.
